# Representation of Multiple Body Parts in the Missing-Hand Territory of Congenital One-Handers

**DOI:** 10.1016/j.cub.2017.03.053

**Published:** 2017-05-08

**Authors:** Avital Hahamy, Scott N. Macdonald, Fiona van den Heiligenberg, Paullina Kieliba, Uzay Emir, Rafael Malach, Heidi Johansen-Berg, Peter Brugger, Jody C. Culham, Tamar R. Makin

**Affiliations:** 1 Department of Neurobiology, Weizmann Institute of Science, Herzl Street, Rehovot 7610001, Israel; 2 Graduate Program in Neuroscience, University of Western Ontario, London, Ontario N6A 5B7, Canada; 3 Brain and Mind Institute, University of Western Ontario, London, Ontario N6A 5B7, Canada; 4 FMRIB Centre, Nuffield Department of Clinical Neuroscience, University of Oxford, Headington, Oxford OX3 9DU, UK; 5 Department of Neurology, Neuropsychology Unit, University Hospital Zurich, Frauenklinikstrasse 26, 8091 Zurich, Switzerland; 6 Department of Psychology, University of Western Ontario, London, Ontario N6A 5B7, Canada; 7 Institute of Cognitive Neuroscience, University College London, London WC1N 3AZ, UK

**Keywords:** amelia, neuroimaging, phantom pain, plasticity, sensorimotor, one-handers

## Abstract

Individuals born without one hand (congenital one-handers) provide a unique model for understanding the relationship between focal reorganization in the sensorimotor cortex and everyday behavior. We previously reported that the missing hand’s territory of one-handers becomes utilized by its cortical neighbor (residual arm representation), depending on residual arm usage in daily life to substitute for the missing hand’s function [1, 2]. However, the repertoire of compensatory behaviors may involve utilization of other body parts that do not cortically neighbor the hand territory. Accordingly, the pattern of brain reorganization may be more extensive [3]. Here we studied unconstrained compensatory strategies under ecological conditions in one-handers, as well as changes in activation, connectivity, and neurochemical profile in their missing hand’s cortical territory. We found that compensatory behaviors in one-handers involved multiple body parts (residual arm, lips, and feet). This diversified compensatory profile was associated with large-scale cortical reorganization, regardless of cortical proximity to the hand territory. Representations of those body parts used to substitute hand function all mapped onto the cortical territory of the missing hand, as evidenced by task-based and resting-state fMRI. The missing-hand territory also exhibited reduced GABA levels, suggesting a reduction in connectional selectivity to enable the expression of diverse cortical inputs. Because the same body parts used for compensatory purposes are those showing increased representation in the missing hand’s territory, we suggest that the typical hand territory may not necessarily represent the hand per se, but rather any other body part that shares the functionality of the missing hand [4].

## Highlights

• Compensatory behavior in one-handers involves utilization of multiple body parts
• Multiple body parts benefit from increased representation in the missing-hand area
• The missing-hand area showed reduced connectional selectivity (lower GABA levels)

## Teaser

Hahamy et al. characterize how daily compensatory behavior associates with changed brain organization in individuals born with one hand. They find that body parts used to substitute hand function show increased representation in the brain territory of the missing hand. These findings offer novel perspectives on brain development and plasticity.

## Results

### Compensatory Behavior in One-Handers Involves Multiple Body Parts

**Figure 1:**
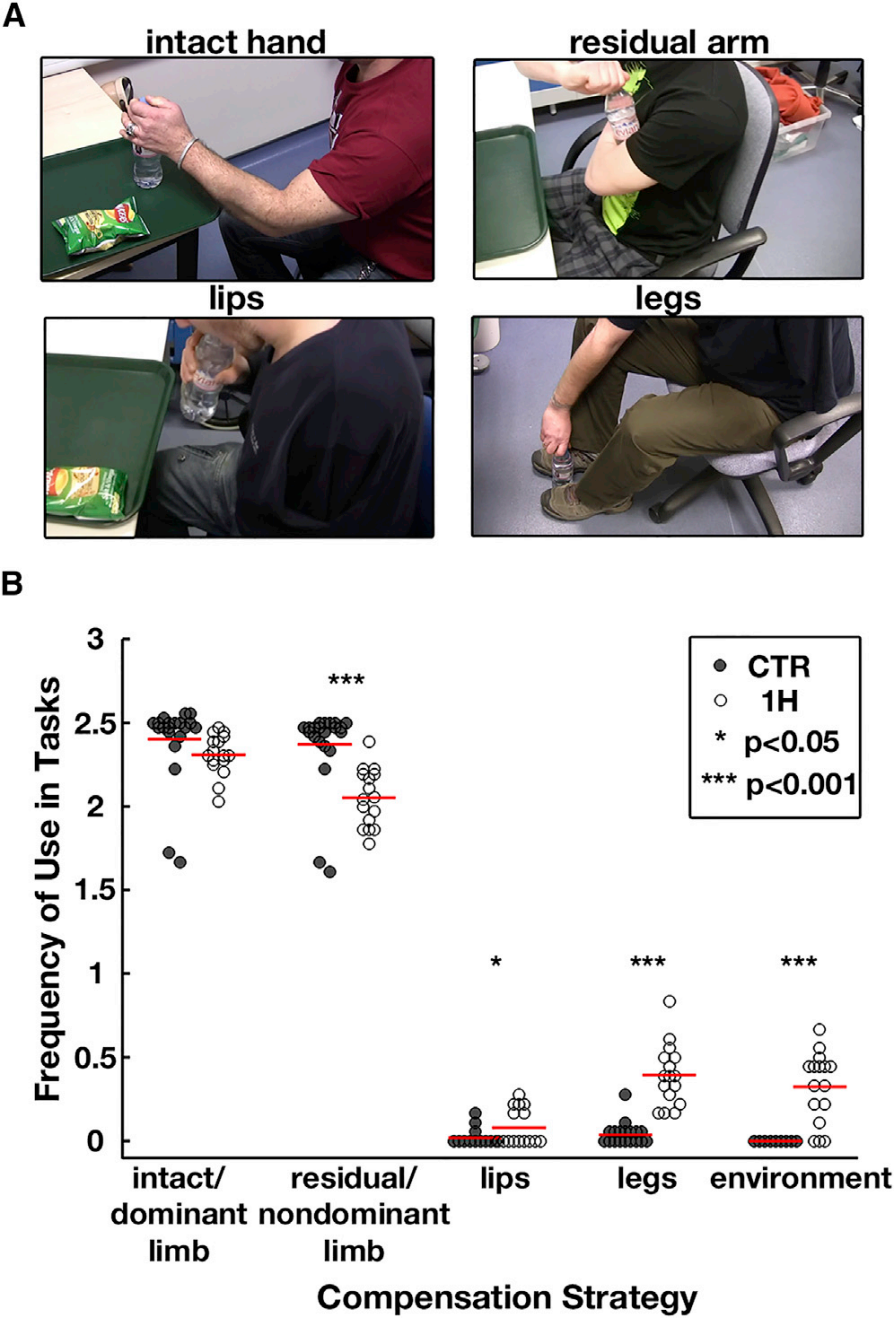
Compensatory Behavior in One-Handers Involves Multiple Body Parts (A) Examples of one-handers opening a bottle during the ecological task. (B) Group comparison of behavioral scores for each body part across tasks. A greater proportion of one-handers (white dots) used their lips, legs, and environment to execute the tasks compared to controls (gray dots). Dots represent individual participants; red lines represent group means. CTR, control participants; 1H, one-handers.

We first characterized compensatory behavioral strategies in congenital one-handers while they performed tasks simulating everyday situations (Figure 1). One-handers mostly relied on their intact hand and residual arm to perform everyday tasks. As expected, they relied on their residual arm less in comparison to controls’ entire nondominant upper limb (hand and arm) (p < 0.001). When compared to controls, one-handers were also more likely to use their lower face (p = 0.02), lower limbs (p < 0.001), and objects in their environment (p < 0.001) to substitute their missing hand’s function, but not their intact hand (see Supplemental Experimental Procedures).

### Increased Activation during Movements of Multiple Body Parts in the Missing-Hand Territory

**Figure 2:**
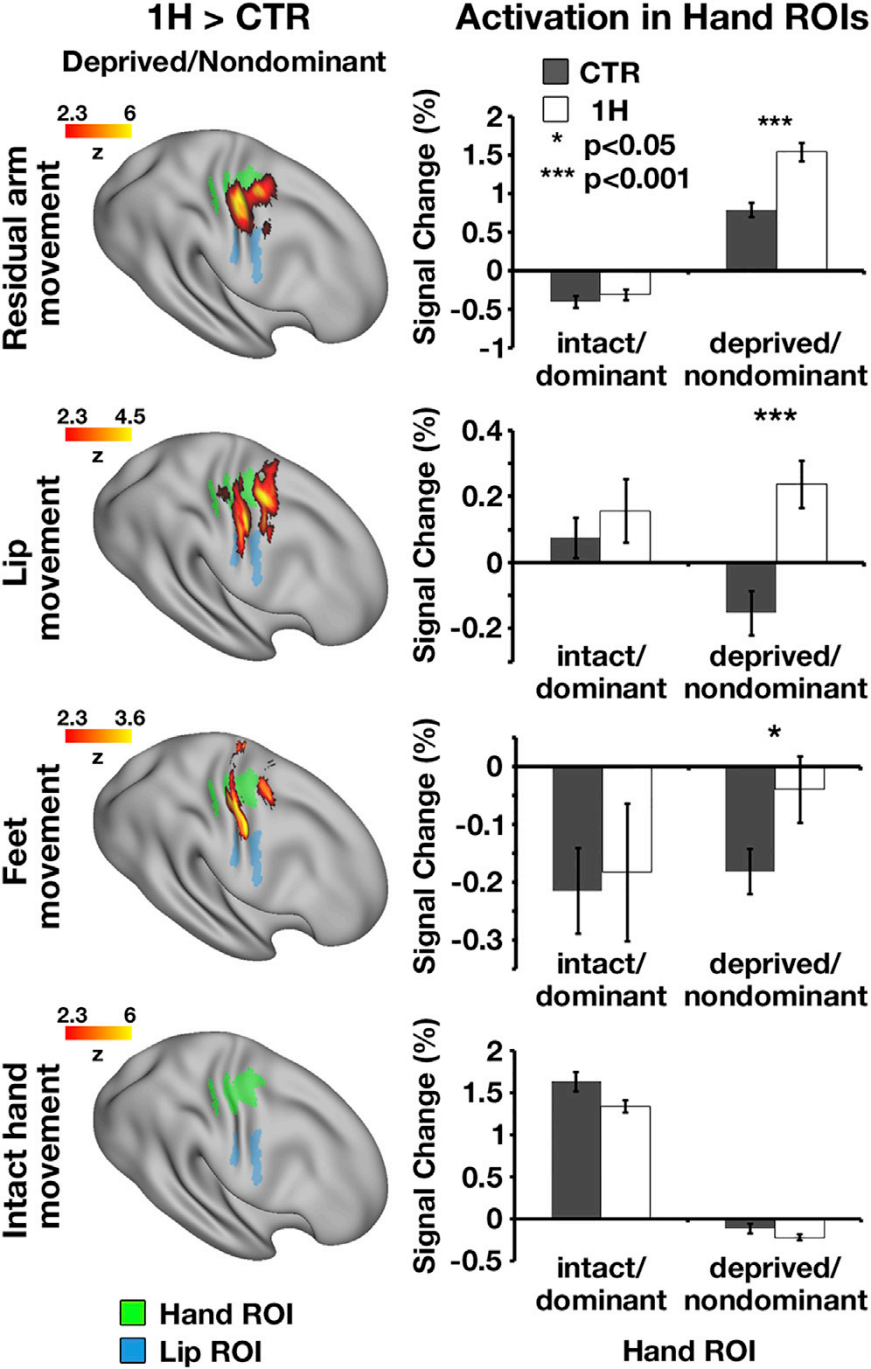
Movements of Body Parts Used for Compensatory Behavior Activate One-Handers’ Missing-Hand Territory Left: group-contrast maps during residual/nondominant arm (one-handers/controls), lips, feet, and intact/dominant hand movements, projected onto an inflated surface of a template brain. In each of the arm, lips, and feet (but not intact hand) conditions, one-handers showed increased activation compared to controls, centered in the missing-hand territory. Green and blue shadings indicate the hand and lip ROIs, respectively. Right: ROI analysis, comparing group activation in the bilateral hand territories. Activation levels in one-handers’ missing-hand territory (white bars) were greater than activations in controls’ nondominant-hand territory (gray bars) in all but the intact-hand condition. 1H, one-handers; CTR, controls; intact/dominant, hemisphere contralateral to the intact/dominant hand; deprived/nondominant, hemisphere contralateral to missing/nondominant hand. Error bars depict SEMs. See also Figures S1 and S3 and Tables S1 and S2.

We next examined activation during movements of those body parts employed for compensatory usage. One-handers’ arm was compared to the controls’ nondominant arm. Whole-brain group contrast maps for movements of the nondominant/residual arm, lips, and feet each showed increased activation centered in the missing-hand territory of one-handers, compared to controls (Figure 2, left panel; Table S1; Figure S1 depicts one-handers and controls’ group maps). This activation expanded beyond the hand area but did not engage the relevant body-part territories, as confirmed in an region of interest (ROI) analysis of the lip and foot areas (Table S2A; see Supplemental Experimental Procedures).

An independent ROI analysis confirmed increased activation in the (putative) missing-hand territory of one-handers during movements of the residual arm (t_(35)_ = 4.93; p < 0.001), lips (t_(36)_ = 3.9; p < 0.001), and feet (t_(36)_ = 2.12; p = 0.04), relative to controls (Figure 2, right panel). When comparing activation in the hand territories across hemispheres and groups, the residual arm (F_(1,35)_ = 17.65, p < 0.001) and lips (F_(1,36)_ = 11.18, p = 0.002), but not the feet (F_(1,36)_ = 1.48, p = 0.23), showed a significant interaction, indicating that increased activation in one-handers is specific to the missing-hand region (Table S2B details activation in the intact-hand region). Movements of the intact hand, which was not overused by one-handers (Figure 1B [2]), did not produce increased activation in one-handers’ missing-hand territory compared to controls (t_(36)_ = 1.47; p = 0.15; Figure 2). A repeated-measures ANOVA with factors group (one-handers, controls), hemisphere (missing, intact), and body part (arm, lips, feet, intact hand) confirmed that reorganization in the missing-hand territory was selective to those body parts used for compensatory purposes (three-way interaction F_(2,34)_ = 5.24, p = 0.01).

### Increased Resting-State Coupling between the Missing-Hand Territory and the Lip and Foot Territories

**Figure 3:**
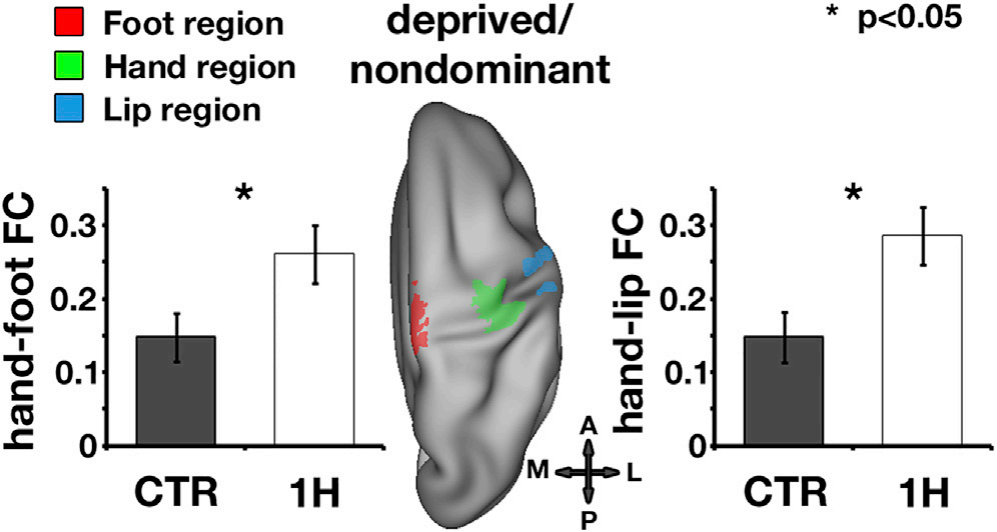
Reorganization Observed in One-Handers’ Resting-State Functional Connectivity ROIs of the foot (red), hand (green), and lip (blue) are projected onto an inflated surface of a template brain, representing the deprived/nondominant hemisphere from a dorsal view. Functional connectivity was increased between the missing-hand territory and the foot (left) and lip (right) ROIs in one-handers (white bars) compared to controls’ nondominant-hand territory (gray bars). Error bars depict SEM. FC, functional connectivity. Other annotations are as in Figure 2. See also Figure S2.

Habitual behaviors have been suggested to be imprinted into resting-state functional connections [1, 5, 6, 7, 8, 9]. We therefore examined whether reorganization in one-handers would also be evident in the functional coupling between the sensorimotor missing/nondominant hand and the lips (contralateral to the missing/nondominant hand), bilateral foot, and intact/dominant hand ROIs using resting-state partial correlations. Compared to controls, one-handers showed increased coupling between the missing-hand ROI and both the lip (t_(38)_ = 2.61; p = 0.01) and foot (t_(38)_ = 2.22; p = 0.03) ROIs (Figure 3; Figures S2A–S2C depict whole-brain functional connectivity results). We also found significant decoupling between one-handers’ bilateral sensorimotor hand territories compared to controls (t_(38)_ = −3.6; p < 0.001), as previously reported [1]. Repeated-measures ANOVA with factors group (one-hander, controls) and connectivity with the missing-hand territory for ROIs (lips, feet, intact hand) revealed a significant interaction (F_(2,37)_ = 8.28, p = 0.001), confirming dissociated connectivity between body-part territories that are used or unused for compensatory purposes and the missing-hand territory.

**Figure 4:**
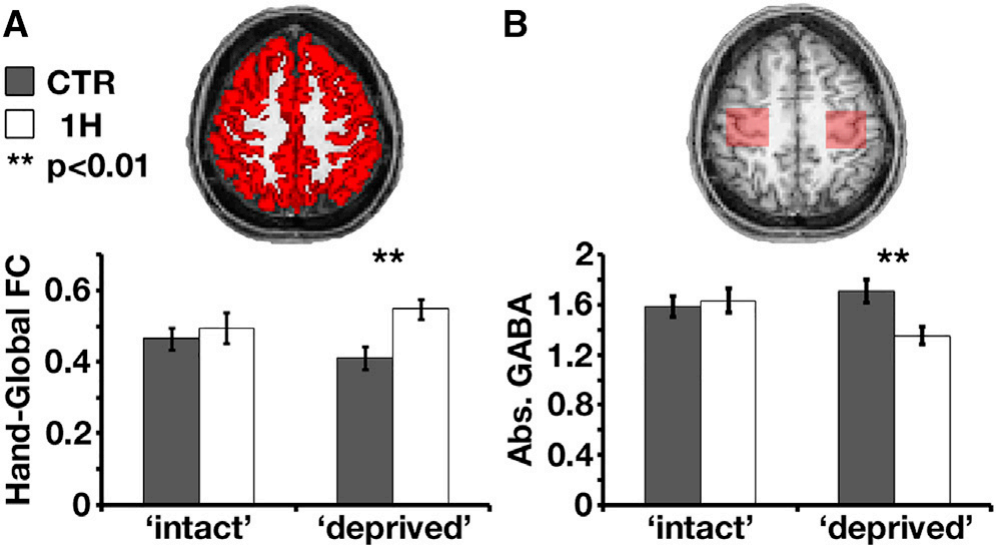
Large-Scale Reorganization in One-Handers, Potential Mechanism (A) Top: resting-state global signal was defined as the averaged time course of gray-matter voxels (red) across the entire brain (illustrated in one participant). Bottom: one-handers’ missing-hand territory (white bars) showed increased coupling with the global signal compared to controls’ nondominant-hand territory (gray bars). (B) Top: illustration of two voxels (red) placed over the bilateral hand knobs, used to extract absolute GABA concentrations using MR spectroscopy. Bottom: one-handers’ missing-hand territory showed reduced GABA concentrations compared to controls’ nondominant-hand territory, suggesting reduced inhibition in the missing-hand territory. FC, functional connectivity. Other annotations are as in Figure 2. Error bars depict SEMs. See also Figure S2.

To test whether the increased connectivity with the missing-hand territory was limited to the sensorimotor cortex, we studied the global signal, defined as the averaged resting-state time course across all gray-matter voxels [10]. One-handers’ missing- hand territory showed greater correlation with the global signal relative to controls (t_(38)_ = 2.86; p = 0.007; Figure 4A), even after regressing out the temporal component representing the sensorimotor network from the global signal (t_(38)_ = 5.73; p < 0.001; see Figure S2D and Supplemental Experimental Procedures). Increased global signal connectivity was specific to the missing-hand territory, as indicated by a significant group by hemisphere interaction (F_(1,38)_ = 12.85, p = 0.001 and F_(1,38)_ = 8.42, p = 0.006, before and after regression of the sensorimotor component from the global signal, respectively). This analysis suggests a weak, albeit widespread, increased coupling between one-handers’ missing-hand territory and the rest of the brain.

### Decreased GABA Concentration in One-Handers’ Missing-Hand Territory

The increased connectivity observed in one-handers’ missing-hand territory may be triggered by reduced inhibitory connections in this region due to a congenital input loss. Decreased connectional selectivity could unmask normally silenced inputs, allowing for increased representation of cortically displaced inputs from other body parts in the missing-hand territory. Indeed, magnetic resonance spectroscopy analysis revealed lower absolute GABA levels in one-handers’ missing-hand territory compared to controls’ nondominant-hand territory (t_(36)_ = 3; p = 0.005; Figure 4B) and a significant group by hemisphere interaction (F_(1,36)_ = 4.83, p = 0.03), supporting our prediction.

### Brain and Behavior Correlations

No significant correlations were found between reorganization measurements and performance on behavioral tasks (see Supplemental Experimental Procedures).

## Discussion

Early studies in congenital one-handers found no lip remapping into the missing-hand territory using passive tactile stimuli [11, 12]. Later studies, using sensorimotor tasks as described here, reported feet remapping into the hand territory in individuals with congenital or early bilateral hand absence, with exceptional abilities to manipulate objects with their feet [13, 14]. Here, we show that multiplex compensatory strategies of typically behaving one-handers is associated with large-scale brain remapping of body representation. Reorganization in the missing-hand territory was observed simultaneously for arm, foot, and lip representations, regardless of cortical distance from the hand territory [15]. Our findings indicate that representations in the missing-hand territory can be flexibly distributed to body parts that share the same functional utility as the absent hand, as will be discussed below. The discrepancy with earlier studies, which used passive tasks and small sample sizes, likely originates from increased activation during active tasks due to expression of multiple inputs into the sensory cortex.

The missing-hand territory showed increased coupling with the global signal, which may provide insight into the process by which this region becomes activated by displaced inputs from the residual arm, lips, and feet. During brain development, the putative hand territory is deprived of peripheral inputs that normally shape its function. Instead, this region may become weakly activated by other, nonspecific inputs [16], as reflected in increased coupling with the global signal. This interpretation is supported by observed GABA reduction in the missing-hand territory, hinting at the unmasking of normally silenced inputs. Consolidation of displaced representations in the missing-hand territory likely depends on Hebbian-like co-activations with descending inputs involved in the canonical function of a hand (e.g., coordination with the other hand). According to the connectivity bias theory [4], the inherent function of a region, and therefore opportunities for its reorganization, will be rooted in its connectivity patterns (as well as sensitivity to task-distinctive features [17]). As compensatory strategies unfold during early childhood, inputs evoked by substituting the missing hand’s function (by the residual arm, lips, and feet) may consolidate more efficiently than non-behaviorally related inputs in the missing-hand territory. Furthermore, other body parts unused for compensatory purposes (the intact hand; see [1, 2, 18]) will not benefit from the missing hand’s resources, regardless of connectional biases (see Supplemental Experimental Procedures for further details). Our findings suggest that the typical hand territory may not necessarily represent the hand per se, but rather any other body part that can mimic the missing hand’s functionality. Together with related findings from visual cortex reorganization in congenitally blind individuals [19], our results suggest that reorganization may be functionally, rather than topologically, restricted. This is in contrast to prominent theories that limit reorganization in the primary somatosensory cortex to cortical neighbors [20, 21, 22].

Lip remapping into the missing-hand territory (as observed using both passive [23] and active [e.g., 24, 25, 26] lip-stimulation paradigms) is considered a major driver of phantom limb pain in amputees [23, 27]. Because congenital one-handers show lip remapping but do not experience phantom pain, our results provide a counter-example to the maladaptive plasticity theory of phantom pain. This and other recent evidence showing typical somatotopy in amputees [15, 28, 29] suggest that the maladaptive plasticity theory should be reconsidered, as well as therapeutic approaches derived from it (e.g., mirror therapy [30]).

Finally, although the same body parts used for compensatory behavior also showed increased activation in the missing-hand territory, no correlations were found between behavior and reorganization. This could be attributed to experimental constraints in capturing variability in compensatory behavior or ecologically valid brain activation. Alternatively, since behavior is likely to alter throughout the course of life, brain reorganization may not reflect compensatory strategies in adulthood, but rather during earlier developmental stages. It is also possible that behavior and brain reorganization are not directly related. For example, the unmasking of otherwise-silenced connections may not necessarily be harnessed to guide behavior [31]. Further research is needed to characterize the relation between brain and behavior throughout the course of life.

## Experimental Procedures

Full experimental procedures are available in the Supplemental Experimental Procedures.

### Participants

Seventeen individuals with congenital unilateral upper-limb deficit (Table S3) and 24 matched two-handed controls were recruited for our study. One one-hander and two controls did not complete the scanning session. Recruitment was carried out with assistance from Opcare (prosthetics providers for National Health Services, UK) in accordance with Oxford University's Medical Sciences inter-divisional research ethics committee (Ref: MSD-IDREC-C2-2014-003). Informed consent and consent to publish was obtained in accordance with ethical standards of the Declaration of Helsinki (1964).

### Behavioral Task

To characterize habitual compensatory behavior, participants completed five tasks, designed to simulate everyday situations (e.g., wrapping a present, handling money, handling cafeteria food). Task performance was video recorded and analyzed offline (see Table S4 for task completion times). Behavior was characterized based on usage of one (or more) of the following body parts during the task: intact/dominant hand and intact/dominant arm (in one-handers and controls, respectively); residual arm and prosthesis (or nondominant hand and arm in controls); mouth and chin; legs and torso; utilization of objects in the environment (see examples in Movie S1.) Performance was assessed based on dependency and frequency of use of each body part for task completion. Two independent raters analyzed the videos offline. An inter-rater reliability assessment using the nonparametric “limits of agreement” [32] established reliability for three of five tasks. Upper-limb scores across one-handers were further validated against questionnaire scores for residual arm usage in daily tasks [2] (Spearman’s rho = −0.81, p < 0.001), confirming the validity of the behavioral results. Behavioral scores were compared between groups using permutation tests.

### Assessment of Brain Reorganization

Each participant underwent one scanning session, involving structural magnetic resonance imaging (MRI), MR spectroscopy, resting-state functional MRI (fMRI), visual task-based fMRI (not reported here), and motor task-based fMRI (see Supplemental Experimental Procedures for acquisition details). Data collected for individuals missing a right hand (n = 4) and for left-handed controls (task: n = 7; rest: n = 8) were horizontally flipped prior to all functional analyses. The proportion of flipped data did not differ between groups (task: χ^2^_(1)_ = 0.21, p = 0.65; rest: χ^2^_(1)_ = 0.32, p = 0.57; see Supplemental Experimental Procedures and Figure S3 for validation of this procedure).

#### fMRI Scans

In the motor task, participants were visually instructed to move their hands (finger flexion/extension), arms (elbow flexion/extension), lips, or feet (bilateral toe movements), as previously detailed [2].

All fMRI data were preprocessed and analyzed using FSL's FEAT (versions 5.0 and 6.0, respectively). Task-based statistical parametric maps were computed for each condition versus resting baseline. Activation maps were thresholded using clusters determined by Z > 2.3 and were family-wise-error corrected using a cluster significance threshold of p < 0.05 with FLAME.

An ROI for the sensorimotor hand territory was defined by averaging the low-level contrasts of intact/dominant hand movements versus rest across the two groups (see Supplemental Experimental Procedures). The putative missing-hand ROI was defined by mirror-flipping the intact/dominant-hand ROI on the x axis [1, 2] (see Supplemental Experimental Procedures for validation of this ROI). ROIs of the arm and lip (in the deprived/nondominant hemisphere) and foot (bilaterally) were defined using a similar procedure. Condition-specific activations within the two hand ROIs were compared between groups using a repeated-measures ANOVA.

In the fMRI resting-state task, participants were instructed to focus on a fixation-cross and let their minds wander. For each participant, the time course of the missing/nondominant-hand ROI was correlated with the time course of each of the lip, foot, and intact-hand ROIs, while partialing out the time courses of the remaining ROIs using MATLAB. Resulting coefficients were compared between groups using repeated-measures ANOVA. In addition, the correlations of the global signal [10] with the hand ROIs for each participant were submitted to a repeated-measures ANOVA.

#### MR Spectroscopy

Data were acquired and preprocessed as described in [33]. Absolute neurochemical concentrations of GABA were extracted from the spectra of each sensorimotor hand region while correcting for voxel tissue content and were compared between groups using repeated-measures ANOVA.

## Author Contributions

A.H. and T.R.M. designed the experiment; S.N.M., F.v.d.H., and T.R.M. designed the behavioral task and collected all data; S.N.M., F.v.d.H., P.K., and U.E. contributed to data analysis, led by A.H.; A.H., R.M., P.B., H.J.-B., J.C.C., and T.R.M. interpreted the results; manuscript writing was led by A.H., J.C.C., and T.R.M.

## Supplemental Information

Document S1. Supplemental Experimental Procedures, Figures S1–S3, and Tables S1–S4

Movie S1. Examples of Tasks and Body Parts Used in the Behavioral Ecological Paradigm, Related to Experimental Procedures

Document S2. Article plus Supplemental Information
