# Supplementary material for: Representation of Multiple Body Parts in the Missing-Hand Territory of Congenital One-Handers
Source: Curr Biol. 2017 May 8;27(9):1350–5. doi: 10.1016/j.cub.2017.03.053 (PMC5434257; doi:10.1016/j.cub.2017.03.053)
Supplement: Document S1. Supplemental Experimental Procedures, Figures S1–S3, and Tables S1–S4 [file mmc1.pdf]

Current Biology, Volume *27*

Supplemental Information

Representation of Multiple Body Parts in the Missing-Hand Territory of Congenital One-Handers

Avital Hahamy, Scott N. Macdonald, Fiona van den Heiligenberg, Paullina Kieliba, Uzay Emir, Rafael Malach, Heidi Johansen-Berg, Peter Brugger, Jody C. Culham, and Tamar R. Makin

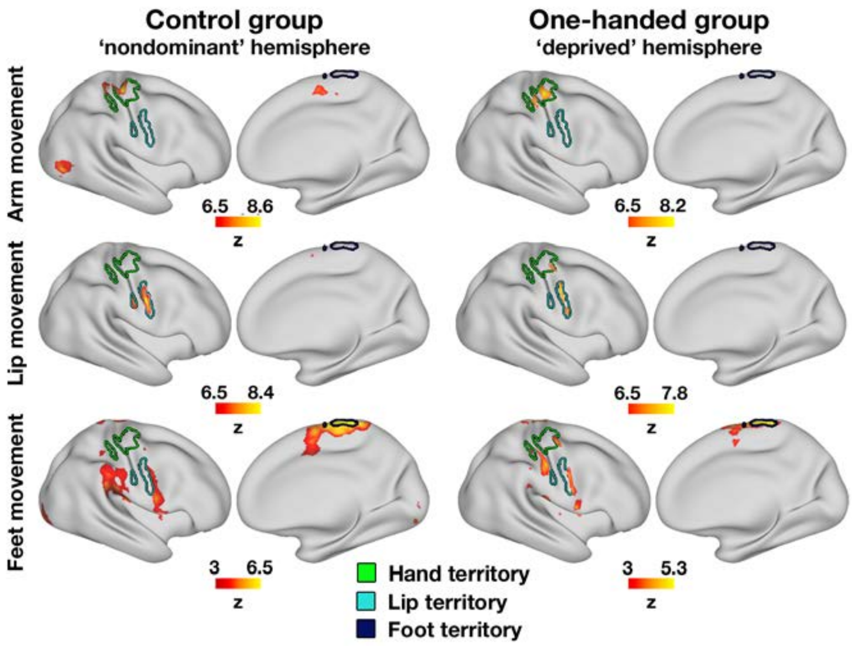

Figure S1. Thresholded group-wise brain activations during execution of simple movements. Related to Figure 2. Group maps of controls (left) and one-handers (right) during the execution of nondominant/residual arm (top row), lips (middle row) and feet (bottom row) movements, against baseline. All activation foci were projected onto the inflated surface of a template brain. Map thresholds were chosen to show distinct peaks along the sensorimotor homunculus and thus visualise qualitative differences between groups (see Fig. 2 for direct group-contrasts using a quantitative approach). In both groups, lip movements activate the lip territory (turquoise contours) and feet movements activate the foot territory (dark blue contours). In addition, all movement conditions activate the missing hand territory in one-handers, but not the nondominant hand region in controls (green contours).

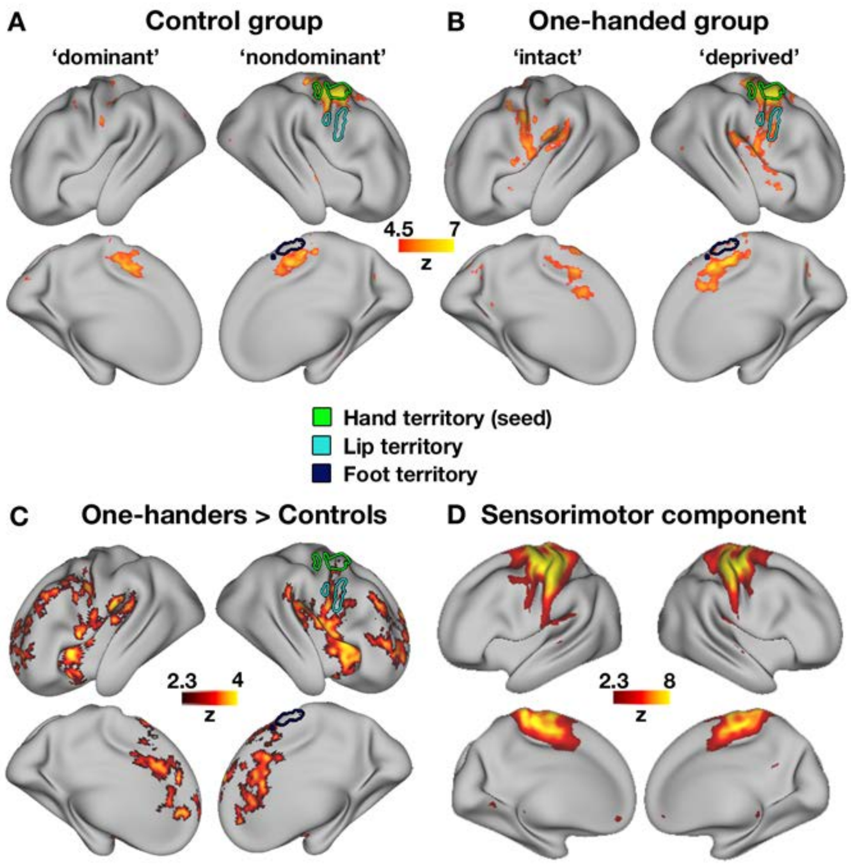

Figure S2. Resting-state analyses maps. Related to Figures 3 and 4. (A) and (B) Thresholded functional connectivity group maps of controls (A) and one-handers (B) based on the nondominant/missing-hand territory seed (green contours), while partialling out the signal from the dominant/intact hand territory. Map thresholds were chosen to highlight those areas showing strong connectivity with the missing-hand territory, rather than all correlated regions. Note that the missing-hand territory shows strong functional connectivity with the lip (turquoise contours) and foot (dark blue contours) territories in one-handers. (C) Between-group contrast of functional connectivity based on the nondominant/missing hand territory seed. Note the global nature of increased functional connectivity in one-handers compared to controls, encompassing also the lip and foot territories. (D) Resting-state independent component of the sensorimotor network. This network was defined using an Independent Component Analysis, ran across all study participants. Intact/dominant; hemisphere contralateral to the intact/dominant hand; deprived/nondominant; hemisphere contralateral to missing/nondominant hand. All connectivity foci were projected onto the inflated surface of a template brain.

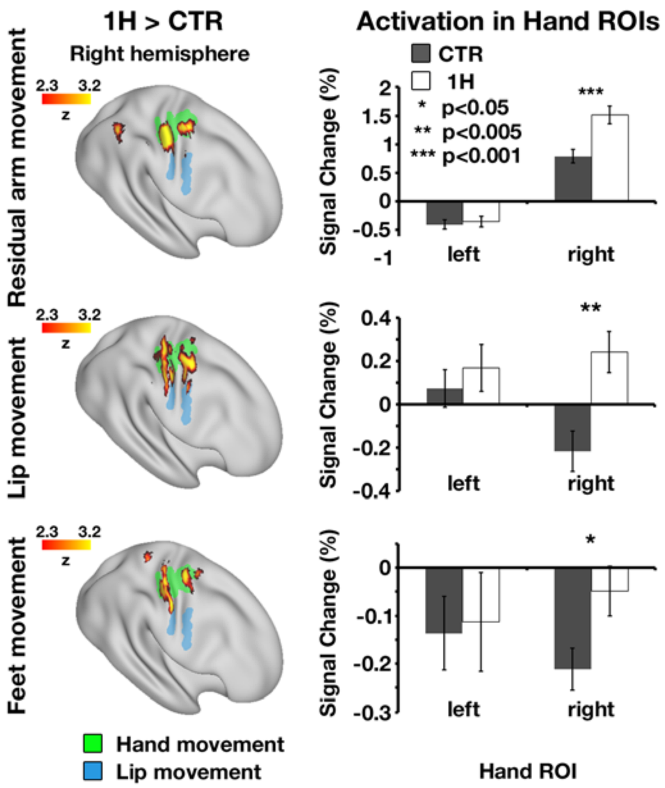

Figure S3. Movements of body parts used for compensatory behaviour activate the right (missing) hand territory of one-handers that are missing their left hand. Related to Figure 2. To avoid the flipping procedure, only individuals with a missing left hand (n=12) and right-handed controls (n=15) were included. Left panel: group contrast maps during movement of the residual/nondominant left arm (one-handers/controls), lips and feet. The green and blue shadings underlying the activation maps indicate the hand and lip ROIs, respectively. In each of these conditions, one-handers showed increased activation compared to controls, centred in the missing-hand territory. All activation foci were projected onto the inflated surface of a template brain. Right panel: ROI analysis, comparing the level of activation (percent signal change) in the bilateral hand territories across groups. Activation levels in one-handers' right-hemisphere (missing-hand) territory (white bars) were greater than activations in controls' right-hemisphere (nondominant-hand) territory (grey bars). 1H, one-handers; CTR, controls; * $p<0.05$; ** $p<0.005$, *** $p<0.001$; left, hemisphere contralateral to the intact/dominant hand; right, hemisphere contralateral to missing/nondominant hand. Error bars depict SEMs.

	# of voxels	Max value	Centre of gravity (X,Y,Z)
**Missing/nondominant hand ROI**	388	-	25,52,64
**Residual/nondominant arm movements**	1031	6.81	22,53,63
**Lip movements**	1095	5	23,54,64
**Feet movements**	700	4.58	23,53,66

Table S1. Cluster statistics for group-contrast statistical maps (one-handers vs. controls). Related to Figure 2. Columns present the cluster size (# of voxels), the value of the maximum ‘intensity’ within the cluster (Max value), the centre of gravity of each cluster given as X/Y/Z values in MNI coordinates. All centres of gravity fell within the region of the missing hand.

	Controls (mean+SEM)	One-handers (mean+SEM)	Statistics
**A. Activation in lip and foot ROIs**			
**Activation in lip ROI during lip movements**	1.65 (0.03)	1.74 (0.03)	$t_{(36)}$= -0.48, p>0.05
**Activation in foot ROI during foot movements**	1.58 (0.04)	1 (0.06)	$t_{(36)}$= 2.03, p=0.05
**B. Activation in the intact/dominant hand ROI**			
**During residual/nondominant arm movements**	-0.41 (0.08)	-0.32 (0.08)	$t_{(35)}$=-0.46, p>0.05
**During lip movements**	0.08 (0.06)	0.16 (0.1)	$t_{(36)}$=-0.46; p>0.05
**During foot movements**	-0.2 (0.07)	-0.18 (0.12)	$t_{(36)}$=-0.81; p>0.05
**During intact hand movements**	1.63 (0.11)	1.34 (0.07)	$t_{(36)}$=1.99; p>0.05

Table S2. Group comparison of activation levels in the lip, foot and intact/dominant hand ROIs. Related to Figure 2. (A) Group comparison of activation levels in lip and foot ROIs during lip and feet movements. Note that although a trend for group difference exists in the feet condition, this difference stems from reduction, rather than increase, in activation in one-handers compared to controls. (B) Group comparison of activation levels in the intact/dominant hand ROI of one-handers/controls, respectively, during movement of different body parts. All activation values were calculated as percent signal change.

Participant	Age	Gender	Affected limb	Level of limb deficiency	Cosmetic Pros. Usage	Functional Pros. Usage	Myo. Pros. Usage	Residual arm usage	Phantom sensation
**PA01**	50	F	L	Below Elbow	4	0	0	0.52	0
**PA02**	53	F	R	Below Elbow	5	1	0	0.43	0
**PA03**	53	M	L	Wrist	0	3	0	0.76	0
**PA04**	26	F	R	Wrist	0	0	0	0.57	0
**PA05**	50	M	L	Above Elbow	1	4	0	0.28	0
**PA06**	29	F	L	Wrist	0	0	0	1	0
**PA07**	39	M	L	Below Elbow	5	0	0	0.46	0
**PA08**	28	F	L	Below Elbow	5	0	0	0.54	0
**PA09**	61	M	L	Wrist	2	0	0	0.3	0
**PA10**	35	F	R	Below Elbow	5	0	0	0.46	0
**PA11**	37	F	R	Below Elbow	5	0	0	0.59	0
**PA12**	42	F	L	Below Elbow	0	0	5	0.54	0
**PA13**	62	F	L	Wrist	5	0	0	0.57	0
**PA14**	26	M	L	Below Elbow	1	0	5	0.69	0
**PA15**	35	M	L	Wrist	0	0	3	0.44	0
**PA16**	39	M	L	Below Elbow	0	2	1	0.78	0
**PA17**	50	F	L	Wrist	1	0	0	0.59	0

Table S3. Demographic details of one-handed individuals. Related to Experimental Procedures. Gender: F=female, M=male; Affected limb: L=left, R=right; Pros. Usage = Prosthetic limb usage (frequency): 0 = never, 1 = rarely, 2 = occasionally, 3 = daily, 4 = more than 4 hours a day, 5 = more than 8 hours a day; Myo.=myoelectric prosthesis; Residual arm usage: based on the Motor Activity Log, ranging between 0 (residual arm never used in everyday tasks) and 1 (residual arm always used in everyday tasks); Phantom sensation: the vividness of sensation, ranging from 0 (no sensation) to 100 (vivid sensation). Participant PA11 did not undergo the ecological behavioural tests, and participant PA13 did not undergo the scanning session.

Time	Wrap a present	Remove money from a wallet	Cafeteria task
**PA01**	01:53	00:31	00:20
**PA02**	02:32	00:40	00:25
**PA03**	01:31	00:50	00:20
**PA04**	02:35	00:38	00:19
**PA05**	03:07	01:11	00:47
**PA06**	NA	00:27	00:18
**PA07**	02:27	00:37	00:21
**PA08**	01:58	00:25	00:18
**PA09**	01:25	01:05	00:28
**PA10**	01:11	00:35	00:19
**PA11**	NA	NA	NA
**PA12**	02:06	00:30	00:24
**PA13**	02:03	00:31	00:27
**PA14**	02:35	00:36	00:20
**PA15**	01:43	00:37	00:13
**PA16**	02:45	00:41	00:23
**PA17**	01:45	00:33	00:30

Table S4. Timing for completion of each validated ecological task per participant (minutes:seconds). Related to Experimental Procedures.

Supplemental Experimental Procedures

Participants

One-handed participants (with either congenital or acquired limb-loss; data from the latter is not reported here) and two-handed controls were invited to take part in a single experimental session, involving questionnaires, behavioural tasks and a magnetic resonance imaging (MRI) scan. Results from a behavioural task using the same group of participants is reported in [S1]. Seventeen individuals with a congenital unilateral upper limb deficit (10 females, age=42.06±11.76, 4 with absent right hand, see Table S3) and 24 two-handed controls (12 females, age=42.67±13.05, 8 left hand dominant) participated in the study. All one-handers suffered from complete or substantial absence of a hand due to interrupted limb formation early in embryonic development (amelia), presented as rudimentary (undeveloped) fingers. In all cases amelia was isolated to a single upper limb, based on volunteers' medical history report. The causes of amelia were not investigated for the purposes of the study. Out of the 17 one-handed participants tested in the current study, 12 had also been tested some 4-5 years earlier in a previous study using a similar paradigm [S2].

Recruitment was carried out with assistance from Opcare (prosthetics providers for National Health Services, UK) in accordance with Oxford University's Medical Sciences inter-divisional research ethics committee (Ref: MSD-IDREC-C2-2014-003). Informed consent and consent to publish was obtained in accordance with ethical standards of the Declaration of Helsinki (1964). One individual from the one-handed group did not participate in the scanning session due to claustrophobia, and an additional one-hander did not participate in the ecological tests. Two control participants did not participate in the scanner motor task due to time constraints, and two additional control participants were excluded from the magnetic resonance (MR) spectroscopy analyses due to technical issues during data acquisition. In addition, data from one one-handed participant with a trans-humeral residual-arm ending (missing elbow) were excluded from the motor-task arm movement analysis.

Questionnaires

Questionnaires were used to collect demographic and clinical details, and assess phantom sensations and pain [as described in S3]. Use of residual arm and prosthesis in one-handers was also assessed with a revised version of the Motor Activity Log [S4], as described previously [S2, S5]. In short, participants with limb absence were requested to rate how frequently they incorporate their residual arm (either directly, or during prosthesis use) in an inventory of typically bimanual daily activities that required varying degrees of motor control. This questionnaire indexing bimanual usage was previously validated using limb acceleration data, collected in ecological settings [S5].

Assessment of compensatory behaviours

To characterise habitual behaviour, all participants were instructed to complete five task sequences designed to simulate every-day situations. Participants were provided with accessories, and instructed regarding specific task-steps that comprised successful completion of each task (see details below). One-handers were encouraged to keep their prosthesis on if they typically carried out these tasks with a prosthesis. Participants started each task seated, but they could stand up if they wished once the task began. The experimenter provided prompts regarding each of the upcoming task-steps to ensure that each task was carried out to completion. Participants were requested to complete the tasks as quickly as possible. No further restrictions were imposed. Task performance was timed and video recorded for offline analyses (see table S4 for completion time details). The entire procedure took approximately

20 minutes. Below we detail the full procedures of the tasks, although note that scoring for only three tasks, as detailed below, met our test-retest reliability criteria.

<u>Task details</u>

Task 1 - Wrap a present: A pair of scissors, scotch tape in a dispenser, a thick roll of wrapping paper, and an empty tea box were placed within reach on a table in front of the participant. Participants were instructed to cover all faces of the box with wrapping paper such that it could be lifted up without unwrapping. This task's rating was validated and used in the final analysis.

Task 2 - Remove money from a wallet: A wallet filled with paper money and plastic cards, an agenda, and a paper clip were placed within reach on a table in front of the participant. Participants were instructed to remove a pre-determined bill (amongst other bills) from the wallet and then attach it to a pre-determined date page in the agenda (corresponding to the participant's birthday) using the paper clip. This task's rating was validated and used in the final analysis.

Task 3 - Write a letter: A pencil (partially sharpened), a small pencil sharpener, a sheet of legal size paper with a bounding box (approximately 3x2 in.) outlined in its centre, and a legal-size envelope were placed within reach on a table in front of the participant. Participants were instructed to sharpen the pencil (3 revolutions), write "brain science is fun" within the bounding box, fold the paper twice along its width to fit in the envelope, and seal the envelope. This task's rating was found unreliable and was therefore excluded from our final analysis.

Task 4 - Fold laundry: A laundry basket containing (from top to bottom) an unfolded (throw) blanket, a large zipped-up rain jacket, and a neuroscience textbook (to weigh down the basket) was placed on the ground to the participants' right. Participants were instructed to stand, pick up the basket, walk around the chair they were seated in, and place the basket on top of the table. They were asked to first fold the blanket (3 folds); then to unzip the rain jacket, put it on, and zip it up. This task's rating was found unreliable and was therefore excluded from our final analysis.

Task 5 – Cafeteria task: A lunch tray supporting a small bag of crisps and a 500-mL bottle of water were placed on the table within reach in front of the participant. Participants were instructed to stand up, carry the tray around their chair (taking care not to drop the food items), place the tray on the table, open the bag of crisps, and open the bottle of water. This task's rating was validated and used in the final analysis.

<u>Rating procedure</u>

Each of the tasks was divided into 3-5 steps required to complete the task. For example, in the cafeteria task, the relevant steps were handling the tray, opening the bag of crisps, and opening the bottle of water. In addition, the means used to execute each step were characterized according to whether the participant accomplished the task using one (or more) of eight body parts (intact/dominant hand, intact/dominant arm, residual arm /nondominant hand and arm, prosthesis, mouth, chin, legs, torso) and/or features or objects within the environment (for example, placing the scotch tape such that it stabilised the wrapping paper while cutting the paper with scissors). For each task-step, an independent rater, who was blinded to the group attribution of each one-handed participant (congenital one-handers/acquired amputees) distributed 10 points across the available 9 means of execution (i.e., the 8 different body parts + the "environment") based on the degree to which each body part or the environment was involved in the completion of the task. This rating was based on the level of dependence and frequency of use of each body part/environment, and not on the

level of sophistication or skill involving the compensatory behaviour. To increase the reliability of ratings, the following means of execution were later grouped by averaging their ratings: intact/dominant hand and intact/dominant arm; residual arm and prosthesis; mouth and chin; legs and torso. Examples of the tasks and body parts used are provided in Movie S1. All ecological data statistical analyses were carried out using in-house Matlab code.

Inter-rater validation

An additional independent observer rated the task performance of ten randomly chosen participants, using the same methodology described above. It is important to note that many means of execution for each participant were given a rating of zero, as many body parts were not used to complete each task. Thus, a simple correlation as a means to assess inter-rater reliability may result in biased estimations of reliability. For this reason, reliability was established using a more conservative methodology. Specifically, the inter-rater bias (difference between raters on each combination of body part and task across participants) was calculated. Since the resulting distribution of differences was found to significantly deviate from normality (Kolmogorov-Smirnov test, $p<0.001$), we employed a nonparametric method to assess inter-rater reliability: the mean bias for each combination of body part and task was tested to evaluate whether it fell within the nonparametric 'limits of agreement', stringently defined as the range between the 10th and 90th percentile of the differences distribution [S6].

This test revealed that the intact/dominant limb ratings of task 3 as well as the residual/nondominant limb ratings of task 4 were unreliable. Since the ratings across body-parts were interdependent (10 points were distributed across all body-parts), the unreliability of a single body-part rating makes the entire task-rating invalid, and thus, only tasks 1, 2 and 5 were submitted to further statistical analyses.

The convergent validity of the upper limb ratings across these three tasks was further validated by assessing the relation of these ratings to previously validated questionnaire scores, which assess the degree of residual arm usage in daily bimanual tasks (hence measuring the level of symmetrical upper limb movement, see Questionnaires section). Laterality in ecological task ratings was calculated as the ratio [(intact−residual)/(intact+residual)], which, as expected, was found to be significantly higher in one-handers compared to controls ($p<0.001$, permutation test, see below). These laterality scores were also highly correlated with the questionnaire scores of residual arm usage (representing behavioural symmetry) across the congenital one-handed participants (Spearman's rho=-0.81, $p<0.001$), further establishing the validity of the ecological task ratings.

The final ratings of each body-part in each participant were submitted to a group-wise permutation test. Participants' labels (one-handers or controls) were shuffled, creating two random experimental groups, and the difference between the group averages was calculated. This procedure was repeated 10,000 times, forming the null distribution. The position of the test's statistic (the difference between the unshuffled group averages) in relation to this distribution was used to calculate the two-tailed p-value. Here and in all further reported statistical analyses, the residual arm/intact hands of one-handers were compared to the nondominant/dominant upper limbs in controls, respectively (note that the proportion of left-handed controls was equivalent to the proportion of left-hand one-handers, as described below). This same permutation procedure was used in all MRI analyses which required permutation testing.

fMRI motor task

To study brain activation evoked by movements of different body parts, we followed the same procedures as previously detailed by Makin et al. [S5]. Participants were visually instructed to move each of their hands (finger flexion/extension), arms (elbow flexion/extension), their feet (bilateral toe movements), or lips, as paced by a visual cue. The usage of an active somatosensory paradigm (e.g. lip movements) was designed to activate both SI and M1. It was previously used to probe consistency in organisation [S3, S7, and see also S8], as well as reorganisation [e.g. S9, S10, S11] in acquired amputees. As such, it is suitable for assessing reorganisation.

One-handers were asked to imagine performing the hand movements with their missing limb (note that none of the one-handers experienced phantom sensations, Table S3). This condition was not submitted to statistical analyses, and was only included to match the experimental design across groups. The protocol consisted of alternating 12-s periods of movement and rest. Each of the six conditions was repeated four times in a semi-counterbalanced order, since due to the number of repetitions and conditions, full counter balancing across conditions could not be achieved [S12]. Participants were trained before the scan on the degree and form of the movements. To confirm that appropriate movements were made at the instructed times, task performance was visually monitored online, and video recordings were made for off-line evaluation.

MR data acquisition and preprocessing

All data were acquired using a 3-Tesla Verio scanner (Siemens, Erlangen, Germany) with a 32-channel head coil. All imaging data (with the exception of MR spectroscopy) were preprocessed using FMRIB's Software Library (FSL; version 5.0).

Anatomy

Anatomical data were acquired using a T1-weighted magnetization prepared rapid acquisition gradient echo sequence (MPRAGE) with the parameters: TR=2040 ms; TE=4.7 ms; flip angle=8°, voxel size=1 mm isotropic resolution.

Motor task

Blood-oxygenation level dependent (BOLD) fMRI was acquired using a multiple gradient echo-planar T2*-weighted pulse sequence, with the parameters: voxel size= 3 mm isotropic, TR=2000 ms; TE=30 ms; flip angle=90°; imaging matrix=64×64; FOV=192 mm axial slices. Forty-six slices with slice thickness of 3 mm and no gap were acquired in the oblique axial plane, covering the whole cortex, with partial coverage of the cerebellum. 300 volumes were collected in the motor task. Additional dummy volumes were acquired before the start of each scan to allow for T1 equilibrium effects. To habituate the participants to the stimuli and thus reduce arousal-related effects during the first experimental block, an additional condition of no interest was acquired in the very beginning of the scan (additional repetition of the first condition), which was collected but included in the GLM model as a nuisance variable.

The following preprocessing steps were applied to each participant's task data: motion correction using FMRIB's Linear Image Registration Tool [MCFLIRT; S13], B0-unwarping, brain extraction using BET [S14], high pass temporal filtering of 150 s, and spatial smoothing using a Gaussian kernel with a FWHM of 5 mm. Functional images were aligned to structural images initially using FMRIB's Linear Image Registration Tool [S13, FLIRT; S15] and then optimized using Boundary-Based Registration [BBR; S16]. Structural images were transformed into MNI space using FMRIB's Nonlinear Image Registration Tool (FNIRT).

Resting state

BOLD fMRI resting state was acquired using a multiband-6 sequence [S17] with the parameters: voxel size=2 mm isotropic, TR=1300 ms; TE=40 ms; flip angle=66°. 72 slices with 2 mm thickness and no slice gap were acquired in the oblique axial plane, covering the whole cortex and most of the cerebellum. 230 volumes were acquired in the resting state run. Additional dummy volumes were acquired before the start of each scan to allow for T1 equilibrium effects. The first dummy volume was saved and later used as a reference for coregistration.

The preprocessing of the resting state fMRI followed the same pipeline as that of the motor task, with high pass temporal filtering of 100 s. In addition, to account for non-neuronal noise that might bias functional connectivity analyses, we regressed out the six motion parameters, as well as the BOLD time series of white-matter and cerebrospinal fluid (CSF) of the unsmoothed data. For this purpose, the T1-weighted structural images were segmented into grey matter, white matter, and CSF, using FSL FAST [S18]. To avoid the inclusion of grey matter voxels in the nuisance masks, these masks included only voxels identified as white-matter/CSF with probability of 1, restricted by an anatomically-based bounding box ($-44<x<42$, $-84<y<42$, $-4<z<34$ for white matter; $-42<x<38$, $-64<y<38$, $-22<z<28$ for CSF), and eroded by a sphere of radius 5 and 2 mm for white-matter and CSF, respectively.

MR Spectroscopy

1H magnetic resonance spectroscopy (MRS) was performed using two (2 cm)^3 voxels, symmetrically centred on the estimated anatomical location of the hand knob of each hemisphere in each participant, acquired consecutively. Off-line evaluation of each of the voxels placement was later conducted, and data from participants with misplaced voxels (two control participants) were discarded from further analysis.

The application of field gradients during scanning deposits heat. As the magnetic properties of the shim elements are temperature-dependent, this could potentially result in field drifts during the imaging sessions. We have accounted for these drifts during acquisition by updating the transmit frequency of the scanner every 32 scans. However, as a precaution and to ensure minimal influence of the heating-induced drifts on the missing/non-dominant hand's spectroscopy readings, the voxel corresponding to the missing/nondominant hand knob in one-handers/controls was acquired first, followed by the intact/dominant hand knob. To further ensure such technical issues did not influence our results, we used post-hoc analyses and found no significant differences in the signal-to-noise ratio between hemispheres, neither in one-handers ($t_{(15)}$=-0.76, p=0.48) nor in controls ($t_{(21)}$=0.51, p=0.61) and no significant group by hemisphere interaction ($F_{(1,36)}$=0.799, p=0.377). The spectral resolution (FWHM) was also comparable across both hand ROIs (one-handers: $t_{(15)}$=0.81, p=0.43; controls: $t_{(21)}$=-1.77,p=0.25) with no significant group by hemisphere interaction found ($F_{(1,36)}$=1.17, p=0.29).

B0 shimming was achieved using a GRESHIM. Spectra were measured with a semi-adiabatic localization by adiabatic selective refocusing (semi-LASER) sequence (TE=30 ms; TR=4 s; 64 averages) with variable power RF pulses with optimized relaxation delays (VAPOR), water suppression and outer volume saturation [S19, S20]. Unsuppressed water spectra acquired from the same volume of interest were used to remove residual eddy current effects and to reconstruct the phased array spectra [S21]. Data were acquired in single shot mode, i.e. each single acquisition was saved separately. Single shot spectra were frequency and phase corrected prior to averaging over 64 scans.

MRS metabolites were quantified with LCmodel [S22] (version 6.3-1B) using a basis set of simulated spectra generated based on previously reported chemical shifts and coupling

constants [S23] using VeSPA simulation library [Versatile Simulation, Pulses and Analysis [S24]]. Simulations were performed with the same RF pulses and sequence timings as that on the 3T system in use. Eight LCModel-simulated macromolecule resonances were included in the analysis at the following positions: 0.91, 1.21, 1.43, 1.67, 1.95, 2.08, 2.25 and 3 ppm [S25]. Absolute neurochemical concentrations of γ-amino-butyric acid (GABA) were extracted from the spectra using water signal as an internal concentration reference. The T2 relaxation of tissue water content [80 ms, S20] was taken into account during the LCmodel fitting, with signal loss due to T2 relaxation of metabolites assumed negligible as the metabolites have long T2 relaxation times compared to the used TE of 28 ms [S20].

To remove the potential impact of the CSF on the metabolites concentration estimate, CSF correction was performed on each voxel. A binary mask of the voxel location was constructed in the same imaging matrix as the structural image and applied to a SPM segmentation to calculate the voxel grey matter, white matter and CSF fractions. Metabolites concentrations were then corrected for the CSF fraction (FCSF) by multiplying the measured values by [1/(1–FCSF)]. This approach assumed CSF containing only negligible amounts of metabolites, as demonstrated with proton spectroscopy on clinical field strength scanners [S26].

To ensure that any differences in metabolite concentrations were not due to differences in the voxel tissue content, metabolite concentrations were calculated relative to an unsuppressed water spectrum acquired from the same voxel, assuming a water content of 72% for white matter, 82% for gray matter and 100% for CSF [S27, S28]. The following equation was then used to correct the water signal individually for each participant:

Water correction = (FGM * GMwater (0.819)) + (FWM * WMwater (0.72)) + (FCSF * CSFwater (1))

Statistical analysis

Statistical analyses of the motor-task data and resting-state whole-brain functional connectivity were performed using FMRIB’s Expert Analysis Tool (FEAT; version 6.0). In house Matlab code (Mathworks, Natick, MA, USA) was used to calculate and test the resting-state partial correlations and global signal connectivity.

Motor fMRI task

Statistical parametric maps were computed for each condition vs. resting baseline as implemented in FSL's FEAT and FLAME, using a double-gamma hemodynamic response function convolved with the experimental model. The six motion parameters were included as regressors of no interest. Data collected for individuals with absent right hands (n=4) and left-handed controls (task: n=7, rest: n=8) were mirror reversed across the mid-sagittal plane prior to all functional analyses. The proportion of flipped data did not differ between groups (task: $\chi^2_{(1)}$=0.21, p=0.65; rest: $\chi^2_{(1)}$=0.32, p=0.57).

To confirm that this procedure did not bias our main results, the results reported in Figure 2 were repeated while accounting for the flipping procedure. We first excluded the 7/22 left-handed control participants and the 4/16 one-handers born without a right hand (resulting in a test sample of 15 right-handed control participants and 12 right-handed one-handers). Thus, no flipped data were included in this analysis. Despite the reduced sample size, a whole brain analysis of activation replicated our main findings, showing significantly increased activation

in the missing hand territory during arm, lip and feet movements of one-handers compared to controls (Figure S3). An ROI analysis of the missing hand cortex showed significantly increased activations in one-handers, compared with controls during arm ($t_{(24)}$=3.69, p=0.0007), lips ($t_{(25)}$=3.29, p=0.002) and feet movements ($t_{(25)}$=2.35, p=0.02). Furthermore, this analysis showed no group differences in the intact hand ROI (arm: $t_{(24)}$=0.4, p=0. 7; lips: $t_{(25)}$=0.7, p=0.5; feet: $t_{(25)}$=0.18, p=0.85), resulting in significant hemisphere by group interactions for arm ($F_{(1,24)}$=12.88, p=0.001) and lips ($F_{(1,25)}$=11.39, p=0.002) (feet: $F_{(1,25)}$=2.3, p=0.14). These results confirm that our original findings were not caused by the flipping procedure. To explore potential involvement of lateralised brain structures in the reported results, we re-ran the group contrast while including all participants, but using the original (non-flipped) activation maps. This analysis revealed no significant group differences in activation.

Regions of interest analysis

A region of interest (ROI) for the sensorimotor representation of the hand was defined by averaging the low-level contrast of intact/dominant hand vs. rest across the two groups (one-handers and controls) (Z>8; centre of gravity in MNI coordinates: x=25, y=52, z=64, 388 functional voxels of $(2\ mm)^3$). To identify the putative missing hand ROI, the intact hand ROI coordinates were mirror-flipped on the x-axis [S2, S5]. The condition-specific contrasts across the two groups were used to define the ROIs for the sensorimotor representations of the lips (contralateral to the nondominant/missing hand, z>8; centre of gravity: x=17, y=60, z=55, 206 voxels) and feet (Z>7.5; centre of gravity: x=44, y=51, z=70, 327 voxels). Note that limitations in both MRI acquisition and analysis resolution precluded reliable separation between the two sensorimotor foot areas. For this reason, a bilateral ROI was defined for the feet. For the same technical reason we were unable to dissociate the somatosensory and motor primary cortices (SI and M1, respectively). Therefore, all body part ROIs are labelled ‘sensorimotor’ cortex.

Mean percept-signal-change activation values were extracted from the single-subject functional data for the intact and missing hand ROIs for all conditions using FSL's Featquery. Planned group comparisons within the missing hand ROI were carried out using two-tailed t-tests. A repeated-measures ANOVA with group as an independent factor and activation levels within the two hand ROIs for each experimental condition as dependent variables was also conducted to determine the specificity of these effects to the missing hand ROI. To confirm that reorganisation was selective to those body-parts used for compensatory purposes in the missing-hand territory, repeated measures ANOVA with factors group, hemisphere and body parts (arm, lip, feet, intact hand) was employed. Activation levels within the lip and foot ROIs during lip and feet movement conditions (respectively) were compared between groups using two-tailed t-tests.

Validation of regions of interest

The putative missing hand ROI was defined by mirror-flipping the inter-group intact/dominant-hand ROI on the x-axis, as practiced in our previous studies[S2, S5]. To ensure that this flipping procedure adequately captured inter-individual differences in hand activation, we tested non-dominant hand activation in the control group. For this purpose, we created a new ROI for the non-dominant hand in controls (using the activation contrast of the non-dominant hand vs. baseline). The contrast map was thresholded at Z=6.7 in order to produce an ROI of a comparable size to the original ROI (comprising 386 voxels showing the strongest activation during non-dominant hand movements). We next extracted the activation level for non-dominant hand movement under both the original (flipped) inter-group ROI and the controls intra-group nondominant-hand ROI. The correlation coefficient across control participants between both ROIs was r(20)=0.91 (r(13)=0.88 for right-handed control

participants, r(5)=0.97 for left-handed control participants, no sub-group difference in correlations: Z=-1.24, p=0.22, Fisher's test), suggesting that the original ROI captured the inter-individual variance for the relevant representation. A paired t-test aimed to quantify any possible difference in activation levels between ROIs found no significant differences ($t_{(21)}$=0.84 p=0.41).

An additional ROI for the sensorimotor representation of the hand was defined by a group conjunction of activity of the intact/dominant hand vs. rest (Z>6.5; centre of gravity in MNI coordinates: x=25, y=52, z=64, 469 functional voxels of 2 mm^3; Note that these centre of gravity coordinates are identical to those obtained from the original ROI). To identify the putative missing hand ROI, the intact hand ROI coordinates were mirror-flipped on the x-axis [S2, S5]. Activation was extracted from under these two ROIs for each participant and each experimental condition. A repeated measures ANOVA with factors group (one-handers, controls), hemisphere (missing, intact) and body part (arm, lip, feet, intact hand) confirmed that reorganisation in the missing-hand territory was selective to those body parts used for compensatory purposes (3-way interaction $F_{(2,34)}$=10.73, p<0.001). Further analysis confirmed increased activation in the missing-hand territory of one-handers during movements of the residual arm ($t_{(35)}$=4.76; p<0.001), lips ($t_{(36)}$=4; p<0.001), and feet ($t_{(36)}$=2.53; p=0.01) relative to controls.

To further confirm the validity of the group ROI, we also repeated our main analysis using individual subject ROIs. For each participant, the 388 voxels showing the strongest activity during the intact/dominant hand condition were chosen from the sensorimotor cortex. These ROIs were then flipped to the missing hand hemisphere to comprise the missing hand ROIs. Activation was extracted from under these two ROIs for each participant and each experimental condition. This analysis revealed increased activation in the missing-hand territory in one-handers during movements of the residual arm ($t_{(35)}$=2.99; p=0.005), lips ($t_{(36)}$=3.22; p=0.003), and feet ($t_{(36)}$=2.19; p=0.03) relative to controls. None of these conditions yielded any difference between groups in the subject-based intact/dominant hand territory (residual arm: $t_{(35)}$=0.98; p=0.33, lips: $t_{(36)}$=1.29; p=0.2, feet: $t_{(36)}$=0.94; p=0.35). This pattern of results echoes our main findings, though the statistical significance is weaker.

Resting state fMRI task

The level of resting-state coupling (functional connectivity) between the missing/nondominant hand ROI and each of the other ROIs - the lip (contralateral to the missing/nondominant hand), bilateral foot and intact/dominant hand - was examined. This was measured for each participant by correlating the time-course of the missing/nondominant hand ROI with the time-course of each other ROI, while partialling out the time-courses of the remaining ROIs (excluding the arm ROI, which largely overlaps the hand ROI [see S5]). The resulting three sets of partial correlation coefficients (missing/nondominant-hand vs. lip, missing/nondominant-hand vs. foot, missing/nondominant-hand vs. intact/dominant-hand ROIs) were Fisher-transformed, and group comparisons were conducted using two-tailed t-tests. Connectivity values were also submitted to repeated-measures ANOVA with factors group (one-handers, controls) and connectivity with the missing hand cortex for ROIs (intact hand, lips, feet).

Whole-brain functional connectivity analysis was performed on the preprocessed data using FEAT, with the signal from the missing/nondominant hand region as a seed. The time-course extracted from the missing-hand ROI is “contaminated” by other components from the sensorimotor system (e.g. the intact hand) that may mask the true hand-specific connectivity. For this reason, the signal from the intact/dominant-hand region was added as nuisance regressor. Group maps were created and corrected as described for the motor task above.

To examine remapping outside the sensorimotor system, the resting-state global signal of each participant was calculated by averaging the time-courses of all grey-matter voxels in

each individual brain [S29]. The global signal was then correlated with the time-courses extracted from each of the two bilateral hand ROIs, and the resulting Fisher-transformed correlation coefficients were submitted to repeated-measures ANOVA with factors group (one-handers, controls) and hemisphere (missing, intact). See below details of regression of the sensorimotor temporal component from the global signal.

Independent component analysis for resting state functional connectivity

To identify the sensorimotor network, data were analysed using MELODIC (Multivariate Exploratory Linear Optimized Decomposition into Independent Components, part of FSL http://www.fmrib.ox.ac.uk/fsl/melodic/). Individual resting-state scans were temporally concatenated across both groups to create a single 4D dataset. To allow for comparable analyses between previous and future studies, we restricted the dimensionality to 25 components [S7, as previously implemented by S30, S31]. This analysis produced an independent component (IC) corresponding with the sensorimotor network, as confirmed by a spatial correlation coefficient of 0.75 to a canonical mask of the sensorimotor network (Figure S2D).

To compare this network across groups, we used the dual regression approach [S30]: The main regressor of interest was the averaged time course underlying the sensorimotor IC, with individual voxels weighted based on their contribution to the group IC. The weighted time-courses of all other ICs were also calculated within each individual participant, using the same procedure, and included as regressors of no interest in a voxel-wise first-level GLM. The output values of this analysis are voxel-wise beta values, representing the strength of connectivity of each voxel with the sensorimotor IC, after accounting for partial contribution of all other ICs.

To identify any regions where connectivity with the sensorimotor network may differ in strength across groups, whole-brain group comparisons were also performed, using permutation tests (as applied in FSL's randomise) and threshold-free cluster estimates. Using this approach we found no evidence for increased connectivity in one-handers compared with controls. This analysis indicates that the sensorimotor IC as a whole was not different across the two groups.

The sensorimotor IC was also used to test whether group-differences in the correlations between the global signal and the signals from the hand ROIs were driven by the sensorimotor network. For each participant, the temporal component of the sensorimotor network was regressed out of the global signal, and the same group-comparison procedure as described above was then repeated using the residual global signal.

MR spectroscopy

To study possible plasticity-related neurochemical alterations in one-handers, absolute GABA levels from the intact- and missing-hand regions were assessed [S32, S33]. These values were submitted to a repeated-measures ANOVA, with factors group (one-handers, controls) and hemisphere (missing, intact).

Brain and Behaviour correlations

Exploratory Pearson correlations were computed between all the following measures: (1) the ecological behaviour scores representing the extent to which one-handers used their lips and feet, (2) the level of activation evoked by the movements of the lips and feet in the missing hand region, measured as percent signal change (3) the level of resting-state partial

correlations between the missing-hand territory and the territories of the lips and feet, and (4) the absolute GABA levels in the missing hand region. All correlations that involved behavioural scores were tested using permutation tests. No significant correlations were revealed.

Reliance on intact hand for compensatory behaviour

In our behavioural analysis we found no significant differences between utilisation of the intact hand in one-handers and the dominant hand in controls during the ecological task (p=0.09, permutation test). This result converges with our previous observation of reduced reliance on the intact hand in congenital one-handers compared to one-handed amputees, based on both subjective (questionnaires) and objective (accelerometry) measurements of lateralised upper limb behavior in daily tasks [S5]. Indeed, the results obtained using the ecological task strongly correlated with the subjective measurements of daily behaviour used in our previous study (see Experimental Procedures section). Together, these findings suggest that rather than relying on their intact hand, congenital one-handers tend to engage multiple body parts (as well as objects in their environment) to substitute for their missing hand’s function. This phenomenon may correspond with a recent observation of mild functional deficits in motor control of one-hander’s intact hand [S34]. It has been hence suggested that direct sensorimotor experience with both hands may be important for the typical development or refinement of motor control of the hand (see Discussion for the connectivity bias theory [S35, S36]). It is therefore possible that due to these mild motor deficits, one-handers prefer relying on other body parts to fulfill the functionality of the missing hand, although further research is needed in order to confirm this hypothesis.

It is also important to note that since the intact hand is not utilised to substitute the missing hand's function, there should be no behavioural drive to induce over-representation of the intact hand in the missing hand territory (unlike the lip and foot representation). As such, even though in the typical brain the connectome of the missing hand territory might be ideally placed for over-representing the intact hand [S37], this is not sufficient to drive reorganisation. Furthermore, we have previously reported [S2], and replicate again here, that the two hand territories show reduced connectivity in congenital one-handers. This finding implies that the connectome of the missing hand territory in one-handers is less likely to support remapping of the intact hand, consistent with our behavioural observations.

Over-activation for multiple body parts outside the missing hand area

While the over-activation in one-handers for the feet and lips conditions is centered on the missing hand area (Table S1), significant activation was also observed inferiolateral to the missing-hand region of interest. Currently, there is an ongoing debate regarding the body part representation laterally neighbouring the hand. Classically, the immediate neighbour of the hand area was considered to be the upper part of the face [S38]. This topography has been subsequently confirmed in humans in multiple neuroimaging studies [S39, S40]. However, recent evidence from new-world monkeys suggests that in SI, the bordering neighbour is the lips [S41]. It is possible that the conflicting evidence do not arise from inter-species differences, but rather from different topographic maps in SI and M1, as recently indicated by Glasser et al. [S42]. Given this current controversy and limited existing evidence, it is difficult to place our findings in a physiological framework, and future research should consider changes in topographic mapping of one-handers outside the immediate border of the hand area.

Supplemental References

S1. van den Heiligenberg, F.M., Yeung, N., Brugger, P., Culham, J.C., and Makin, T.R. (2017). Adaptable Categorization of Hands and Tools in Prosthesis Users. Psychological Science, 0956797616685869.

S2. Hahamy, A., Sotiropoulos, S.N., Henderson Slater, D., Malach, R., Johansen-Berg, H., and Makin, T.R. (2015). Normalisation of brain connectivity through compensatory behaviour, despite congenital hand absence. eLife *4*.

S3. Makin, T.R., Scholz, J., Filippini, N., Slater, D.H., Tracey, I., and Johansen-Berg, H. (2013). Phantom pain is associated with preserved structure and function in the former hand area. Nature Communications *4*.

S4. Uswatte, G., Taub, E., Morris, D., Light, K., and Thompson, P.A. (2006). The Motor Activity Log-28: assessing daily use of the hemiparetic arm after stroke. Neurology *67*, 1189-1194.

S5. Makin, T.R., Cramer, A.O., Scholz, J., Hahamy, A., Slater, D.H., Tracey, I., and Johansen-Berg, H. (2013). Deprivation-related and use-dependent plasticity go hand in hand. Elife *2*.

S6. Bland, J.M., and Altman, D.G. (1999). Measuring agreement in method comparison studies. Statistical methods in medical research *8*, 135-160.

S7. Makin, T.R., Filippini, N., Duff, E.P., Henderson Slater, D., Tracey, I., and Johansen-Berg, H. (2015). Network-level reorganisation of functional connectivity following arm amputation. Neuroimage *114*, 217-225.

S8. Raffin, E., Richard, N., Giraux, P., and Reilly, K.T. (2016). Primary motor cortex changes after amputation correlate with phantom limb pain and the ability to move the phantom limb. Neuroimage *130*, 134-144.

S9. Lotze, M., Flor, H., Grodd, W., Larbig, W., and Birbaumer, N. (2001). Phantom movements and pain. An fMRI study in upper limb amputees. Brain *124*, 2268-2277.

S10. MacIver, K., Lloyd, D.M., Kelly, S., Roberts, N., and Nurmikko, T. (2008). Phantom limb pain, cortical reorganization and the therapeutic effect of mental imagery. Brain *131*, 2181-2191.

S11. Foell, J., Bekrater-Bodmann, R., Diers, M., and Flor, H. (2014). Mirror therapy for phantom limb pain: brain changes and the role of body representation. Eur J Pain *18*, 729-739.

S12. Abbuhl, R., Gass, S., and Mackey, A. (2014). 7 Experimental research design. Research Methods in Linguistics, 116.

S13. Jenkinson, M., Bannister, P., Brady, M., and Smith, S. (2002). Improved optimization for the robust and accurate linear registration and motion correction of brain images. NeuroImage *17*, 825-841.

S14. Smith, S.M. (2002). Fast robust automated brain extraction. Hum Brain Mapp *17*, 143-155.

S15. Jenkinson, M., and Smith, S. (2001). A global optimisation method for robust affine registration of brain images. Med Image Anal *5*, 143-156.

S16. Greve, D.N., and Fischl, B. (2009). Accurate and robust brain image alignment using boundary-based registration. NeuroImage *48*, 63-72.

S17. Ugurbil, K., Xu, J., Auerbach, E.J., Moeller, S., Vu, A.T., Duarte-Carvajalino, J.M., Lenglet, C., Wu, X., Schmitter, S., Van de Moortele, P.F., et al. (2013). Pushing spatial and temporal resolution for functional and diffusion MRI in the Human Connectome Project. Neuroimage *80*, 80-104.

S18. Zhang, Y., Brady, M., and Smith, S. (2001). Segmentation of brain MR images through a hidden Markov random field model and the expectation-maximization algorithm. IEEE Trans Med Imaging *20*, 45-57.

S19. Oz, G., and Tkac, I. (2011). Short-echo, single-shot, full-intensity proton magnetic resonance spectroscopy for neurochemical profiling at 4 T: validation in the cerebellum and brainstem. Magn Reson Med *65*, 901-910.

S20. Deelchand, D.K., Adanyeguh, I.M., Emir, U.E., Nguyen, T.M., Valabregue, R., Henry, P.G., Mochel, F., and Oz, G. (2015). Two-site reproducibility of cerebellar and brainstem neurochemical profiles with short-echo, single-voxel MRS at 3T. Magn Reson Med *73*, 1718-1725.

S21. Natt, O., Bezkorovaynyy, V., Michaelis, T., and Frahm, J. (2005). Use of phased array coils for a determination of absolute metabolite concentrations. Magn Reson Med *53*, 3-8.

S22. Provencher, S.W. (1993). Estimation of metabolite concentrations from localized in vivo proton NMR spectra. Magn Reson Med *30*, 672-679.

S23. Govindaraju, V., Young, K., and Maudsley, A.A. (2000). Proton NMR chemical shifts and coupling constants for brain metabolites. NMR Biomed *13*, 129-153.

S24. Soher, B., Semanchuk, P., Todd, D., Steinberg, J., and Young, K. (2011). VeSPA: integrated applications for RF pulse design, spectral simulation and MRS data analysis. In Proceedings of the 19th Annual Meeting of ISMRM, Montreal, Canada. p. 1410.

S25. Schaller, B., Xin, L., Cudalbu, C., and Gruetter, R. (2013). Quantification of the neurochemical profile using simulated macromolecule resonances at 3 T. NMR Biomed *26*, 593-599.

S26. Lynch, J., Peeling, J., Auty, A., and Sutherland, G.R. (1993). Nuclear magnetic resonance study of cerebrospinal fluid from patients with multiple sclerosis. Can J Neurol Sci *20*, 194-198.

S27. Gelman, N., Ewing, J.R., Gorell, J.M., Spickler, E.M., and Solomon, E.G. (2001). Interregional variation of longitudinal relaxation rates in human brain at 3.0 T: relation to estimated iron and water contents. Magn Reson Med *45*, 71-79.

S28. Randall, L.O. (1938). Chemical topography of the brain. Journal of Biological Chemistry *124*, 481-488.

S29. Hahamy, A., Calhoun, V., Pearlson, G., Harel, M., Stern, N., Attar, F., Malach, R., and Salomon, R. (2014). Save the global: global signal connectivity as a tool for studying clinical populations with functional magnetic resonance imaging. Brain Connect *4*, 395-403.

S30. Filippini, N., MacIntosh, B.J., Hough, M.G., Goodwin, G.M., Frisoni, G.B., Smith, S.M., Matthews, P.M., Beckmann, C.F., and Mackay, C.E. (2009). Distinct patterns of brain activity in young carriers of the APOE-epsilon4 allele. Proceedings of the National Academy of Sciences of the United States of America *106*, 7209-7214.

S31. Filippini, N., Nickerson, L., Beckmann, C.F., Ebmeier, K.P., Frisoni, G.B., Matthews, P.M., Smith, S.M., and Mackay, C.E. (2012). Age-related adaptations of brain function during a memory task are also present at rest. Neuroimage *59*, 3821-3828.

S32. Near, J., Andersson, J., Maron, E., Mekle, R., Gruetter, R., Cowen, P., and Jezzard, P. (2013). Unedited in vivo detection and quantification of gamma-aminobutyric acid in the occipital cortex using short-TE MRS at 3 T. NMR Biomed *26*, 1353-1362.

S33. Sampaio-Baptista, C., Filippini, N., Stagg, C.J., Near, J., Scholz, J., and Johansen-Berg, H. (2015). Changes in functional connectivity and GABA levels with long-term motor learning. Neuroimage *106*, 15-20.

S34. Philip, B., Buckon, C., Sienko, S., Aiona, M., Ross, S., and Frey, S. (2015). Maturation and experience in action representation: Bilateral deficits in unilateral congenital amelia. Neuropsychologia *75*, 420-430.

S35. Mahon, B.Z., and Caramazza, A. (2011). What drives the organization of object knowledge in the brain? Trends Cogn Sci *15*, 97-103.

S36. Heimler, B., Striem-Amit, E., and Amedi, A. (2015). Origins of task-specific sensory-independent organization in the visual and auditory brain: neuroscience evidence, open questions and clinical implications. Curr Opin Neurobiol *35*, 169-177.

S37. Stark, D.E., Margulies, D.S., Shehzad, Z.E., Reiss, P., Kelly, A.M., Uddin, L.Q., Gee, D.G., Roy, A.K., Banich, M.T., Castellanos, F.X., et al. (2008). Regional variation in interhemispheric coordination of intrinsic hemodynamic fluctuations. The Journal of neuroscience : the official journal of the Society for Neuroscience *28*, 13754-13764.

S38. Penfield, W., and Rasmussen, T. (1950). The cerebral cortex of man; a clinical study of localization of function.
S39. Zeharia, N., Hertz, U., Flash, T., and Amedi, A. (2015). New whole-body sensory-motor gradients revealed using phase-locked analysis and verified using multivoxel pattern analysis and functional connectivity. J Neurosci *35*, 2845-2859.
S40. Moulton, E.A., Pendse, G., Morris, S., Aiello-Lammens, M., Becerra, L., and Borsook, D. (2009). Segmentally arranged somatotopy within the face representation of human primary somatosensory cortex. Human brain mapping *30*, 757-765.
S41. Liao, C.C., Reed, J.L., Kaas, J.H., and Qi, H.X. (2016). Intracortical connections are altered after long-standing deprivation of dorsal column inputs in the hand region of area 3b in squirrel monkeys. J Comp Neurol *524*, 1494-1526.
S42. Glasser, M.F., Coalson, T.S., Robinson, E.C., Hacker, C.D., Harwell, J., Yacoub, E., Ugurbil, K., Andersson, J., Beckmann, C.F., Jenkinson, M., et al. (2016). A multi-modal parcellation of human cerebral cortex. Nature *536*, 171-178.
