# Supplementary figures and images for: Representation of Multiple Body Parts in the Missing-Hand Territory of Congenital One-Handers
Source: Curr Biol. 2017 May 8;27(9):1350–5. doi: 10.1016/j.cub.2017.03.053 (PMC5434257; doi:10.1016/j.cub.2017.03.053)

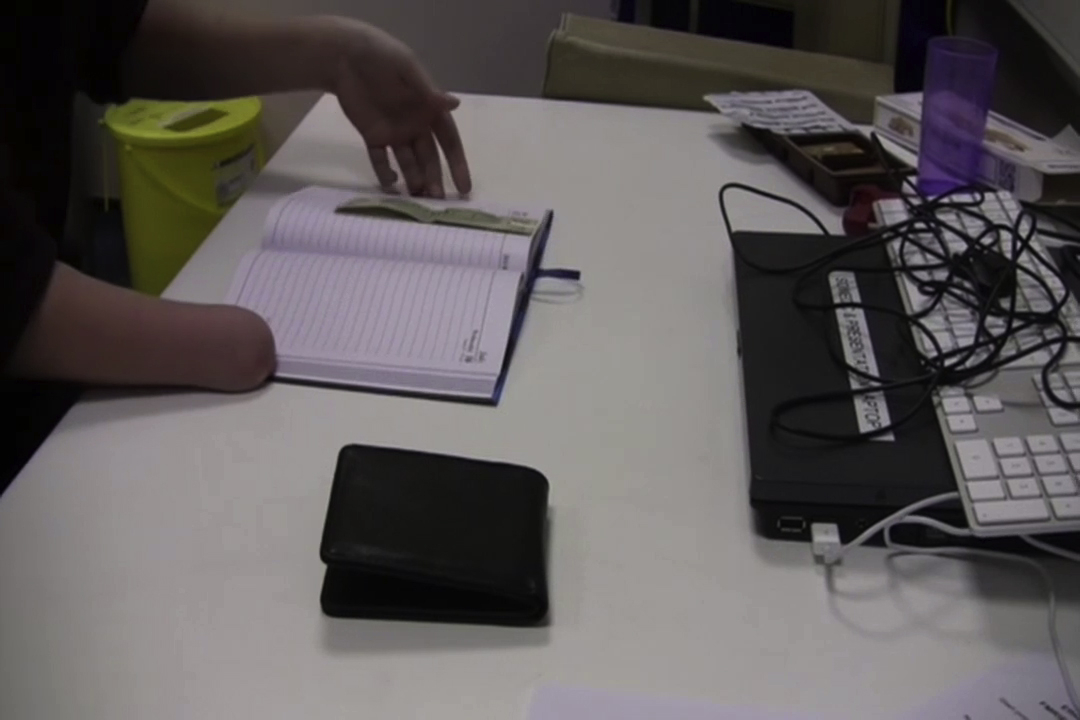

Supplement: Movie S1. Examples of Tasks and Body Parts Used in the Behavioral Ecological Paradigm, Related to Experimental Procedures [file mmc2.jpg]
